# Mitigating skin tone bias in linear array *in vivo* photoacoustic imaging with short-lag spatial coherence beamforming

**DOI:** 10.1016/j.pacs.2023.100555

**Published:** 2023-09-11

**Authors:** Guilherme S.P. Fernandes, João H. Uliana, Luciano Bachmann, Antonio A.O. Carneiro, Muyinatu A. Lediju Bell, Theo Z. Pavan

**Affiliations:** a Department of Physics, FFCLRP, University of Sao Paulo, Brazil; b Department of Electrical and Computer Engineering, Johns Hopkins University, USA; c Department of Biomedical Engineering, Johns Hopkins University, USA; d Department of Computer Science, Johns Hopkins University, USA

**Keywords:** 0000, 1111, Photoacoustic imaging, Clutter artifact, Melanin, Short-lag spatial coherence, Skin pigmentation, Ultrasound, Individual typology angle, Fitzpatrick scale

## Abstract

Photoacoustic (PA) imaging has the potential to deliver non-invasive diagnostic information. However, skin tone differences bias PA target visualization, as the elevated optical absorption of melanated skin decreases optical fluence within the imaging plane and increases the presence of acoustic clutter. This paper demonstrates that short-lag spatial coherence (SLSC) beamforming mitigates this bias. PA data from the forearm of 18 volunteers were acquired with 750-, 810-, and 870-nm wavelengths. Skin tones ranging from light to dark were objectively quantified using the individual typology angle (ITA°). The signal-to-noise ratio (SNR) of the radial artery (RA) and surrounding clutter were measured. Clutter was minimal (e.g., −16 dB relative to the RA) with lighter skin tones and increased to −8 dB with darker tones, which compromised RA visualization in conventional PA images. SLSC beamforming achieved a median SNR improvement of 3.8 dB, resulting in better RA visualization for all skin tones.

## 1 Introduction

Photoacoustic (PA) imaging is a modality based on the PA effect, where the energy deposited by electromagnetic wave absorption causes a thermal expansion followed by the emission of broadband acoustic waves over the MHz frequency range [1]. As a result, it is straightforward to use conventional MHz-range clinical ultrasound (US) transducers to detect these waves [2]. In doing so, PA imaging benefits from employing the same detector as in widely available diagnostic US imaging techniques. In addition, the intrinsic capability of obtaining molecular and functional information, has empowered PA imaging as a rapidly growing modality [3]. Thus, several clinical applications are being investigated [4], [5], including applications in cardiovascular diseases [6], joint arthritis [7], [8], [9], cancer [10], [11], breast imaging [12], and surgical guidance [13], [14], [15].

PA imaging using linear array transducers has traditionally adopted the reflection mode acquisition (also known as epi-photoacoustic imaging), where optical fibers are attached to the US transducer to illuminate the tissue through the skin [16]. While this illumination mode is necessary for non-invasive imaging applications, it suffers from two common limitations. First, the illumination beam strikes the skin, which can be characterized by high optical absorption coefficient in the visible and near-infrared range, due to the melanin content in the epidermis layer [17], [18]. This light absorption can considerably affect the illumination efficiency, particularly for deep-located structures in the imaging plane. Second, the PA waves generated due to light absorption at the skin surface can cause the skin to act as an US emitter, transmitting sound waves beyond the skin surface, which are then reflected by typical US scatterers within tissue, thus creating considerable artifacts in PA images [19]. In particular, PA waves generated by strong optical absorbers at the skin surface interact with the surrounding media and travel back to the US transducer, introducing an artifact known as acoustic clutter. Previous studies have reported the presence of acoustic clutter artifacts in PA images when either bright-field or dark-field illumination schemes were used [19], [20]. This artifact increases the background signal level and deteriorates the signal-to-noise ratio (SNR), contrast, and imaging depth [21]. Therefore, it is important to understand how the epidermal melanin content impacts PA images acquired when light illuminates the skin to image underlying targets.

Skin optical absorption coefficients are mainly dictated by melanin content in the epidermis layer. Therefore, increased epidermal melanin content results in greater skin optical absorption coefficients [18]. It is expected that PA images from darker skinned individuals will suffer from both lower light fluence delivered within the imaging plane and higher levels of clutter artifacts. These limitations are assumed to be the two main contributing factors to compromise the resulting PA image quality obtained with darker skinned individuals, introducing bias within PA imaging based on the epidermal melanin content [20], [22], [23], [24]. In addition, Mantri et al. [20] verified that skin tone can affect PA-based oxygen saturation estimation. Similarly, Li et al. [22] showed that the total blood volume calculated for individuals with high melanin content needs to be compensated. However, the relationships among skin tone, clutter level, and resulting image quality have not yet been quantified and are therefore not fully understood.

Skin tone is typically classified according to the Fitzpatrick skin type (FST) scale, which is based on self-reported erythema sensitivity and ability to tan [25]. Although FST is widely adopted in dermatology, it can be inaccurate and is not consistently used [26]. Therefore, an objective and quantitative method of skin tone classification is preferred [27]. This can be achieved with measurements based on the CIE $L^{*}a^{*}b^{*}$ color system, using devices such as colorimeters and spectrophotometers. In this case, $L^{*}$ is the luminance with values ranging from 0 (black) to 100 (white), and $a^{*}$ and $b^{*}$ indicates the red–green and yellow–blue components, respectively [28]. From $L^{*}a^{*}b^{*}$ values, the individual typology angle (ITA°) can be calculated to assess skin constitutive pigmentation level and, therefore, classify the skin tone as *very light*, *light*, *intermediate*, *tan*, *brown*, or *dark* [28]. Furthermore, ITA° is well correlated with the epidermal melanin content [29], [30].

Previous studies have shown that the short-lag spatial coherence (SLSC) beamformer is capable of reducing clutter in PA images and can improve image quality in low light fluence and noisy environments [31]. SLSC computes the spatial coherence between received signals at different transducer element separations (i.e., spatial lags), then sums across the resulting coherence values in the short spatial lag region to generate an image [32]. Because clutter sources have low spatial coherence, PA clutter artifacts tend to be mitigated in SLSC images, presenting as lower SLSC pixel values [33], [34], [35], [36], [37]. Therefore, SLSC beamforming is expected to reduce skin tone bias in PA imaging by reducing the clutter artifacts that compromise image interpretation and also by improving the visualization of targets subject to low optical fluence [24].

In this paper, the impact of the epidermal melanin content on conventional amplitude-based PA images as well as the capability of SLSC beamforming to reduce clutter artifact was investigated with data acquired from the forearm of 18 volunteers with various skin pigmentation levels. In addition, SNR, clutter level, skin signal, and radial artery (RA) signal were analyzed as functions of ITA° and optical wavelength to provide the first known quantitative characterizations of skin tone, clutter level, and image quality relationships for both SLSC and more conventional PA imaging. The remainder of this paper is organized as follows. Section 2 describes the imaging system used in the experiments, the volunteers’ skin tone classifications, data acquisition, and associated data analysis, including evaluation of PA signals from different tissues and with different clutter levels. Section 3 describes the results, Section 4 discusses the implications of the presented results, and Section 5 summarizes the main conclusions of this work.

## 2 Materials and methods

### 2.1 Imaging system

The imaging system consisted of a Nd:YAG laser (Brilliant B, Quantel Laser) coupled to an optical parametric oscillator (MagicPRISM, Opotek) and a trifurcated optical fiber bundle (77,536, Newport) attached to a linear array transducer (L14-5/38, Ultrasonix) with a center frequency 7.2 MHz and 128 elements. The transducer was connected to a commercial ultrasound machine (SonixOP, Ultrasonix) with a parallel acquisition module (SonixDAQ, Ultrasonix) operating with a sampling frequency of 40 MHz.

The energy level of each pulse was recorded, using a fraction of the laser beam. Based on this recording, each photoacoustic image acquisition was normalized by the pulse energy value to compensate for the pulse-to-pulse energy variation. The recording for each image revealed that the optical fluence at the skin surface was maintained between 2.5 mJ/cm${}^{2}$ and 3.6 mJ/cm${}^{2}$ for all experiments (i.e., well below the 25.2 mJ/cm${}^{2}$, 33.2 mJ/cm${}^{2}$, and 43.75 mJ/cm${}^{2}$ laser safety limits reported for skin at the investigated laser wavelengths of 750 nm, 810 nm, and 870 nm, respectively [38], although this limit does not specify the associated skin tone).

**Fig. 1:**
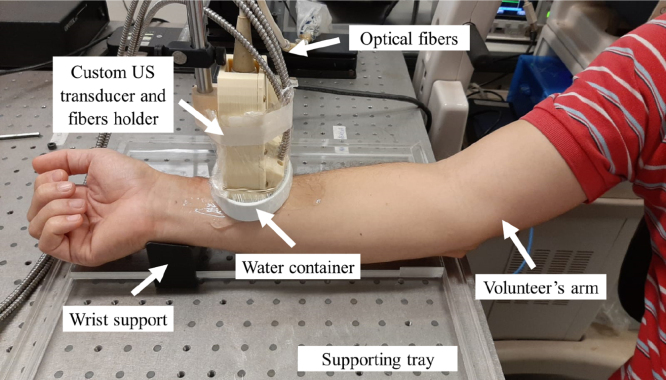
Experimental setup used for the experiments.

A custom 3D-printed support (ZMorph 2.0 SX, ZMorph) was designed to place the optical fiber bundle tips along a line parallel to the lateral direction of the US transducer with an inclination angle of 30° and a distance of 17 mm from the transducer elements’ center. To improve the imaging plane illumination, a pad was attached to the end of the support, distancing the set transducer/fiber tips 10 mm from the skin surface. The pad had an opening window for sound and light propagation, which was sealed with a thin plastic film and filled with water for acoustic coupling between the transducer and the forearm (Fig. 1).

Previous *in vivo* and phantom studies were conducted in our laboratory, with a similar experimental setup, to evaluate the performance of the customized PA imaging system [16], [39], [40]. To assess the stability of the system, PA images were acquired from a wall-less vascular phantom [39] at hourly intervals, over a period of 5 h. Between each data acquisition, we removed the transducer and coupling pad from the phantom, then replaced these items in contact to resume imaging. Styrene-ethylene/butylene-styrene (SEBS) copolymer-in-mineral oil was used as the base material for the phantom [41]. Glycerol was used to further adjust the acoustic properties [42] and TiO${}_{2}$ to increase optical scattering. The vessel was filled with CuSO${}_{4}$ solution to mimic the optical absorption of blood. For detailed information about the production and characterization of the wall-less vascular phantom the reader is referred to [39]. Supplementary material Fig. S1 shows the stability of signal amplitude averaged over selected regions of interest (ROIs) within the vessel and background.

### 2.2 Skin tone classification

Prior to the acquisition of PA images, a depilatory cream (Needs Depil Aloe Vera) was applied on the imaging region (anterior forearm of each volunteer) to minimize the presence of hair at the skin surface. To quantify the volunteer skin pigmentation level, the $L^{*}a^{*}b^{*}$ values were measured using a commercial colorimeter (Delta Vista 450G) and ITA° was calculated using the following equation [28], [43], [44]:

(1) $$\text{ITA}{}^{\circ}=\tan^{-1}\left(\frac{L^{*}-50}{b^{*}}\right)\cdot \left(\frac{180{}^{\circ}}{\pi }\right)$$

The calculated ITA° was then used to classify volunteer skin tone, as defined in [28], [43], [44] and summarized in Table 1.

**Table 1:** Skin tone classification [28], [43], [44].

Grading	ITA	Skin tone category
I	>55°	Very light
II	>41°and≤55°	Light
III	>28°and≤41°	Intermediate
IV	>10°and≤28°	Tan
V	≥−30°and≤10°	Brown
VI	<−30°	Dark

### 2.3 Data acquisition

For the experiment, the subject remained seated and rested the arm on a tray containing a 3D-printed ergonomic wrist support to minimize movement during data acquisition (Fig. 1). B-mode ultrasound imaging was used to place the RA within the imaging plane. The supplementary video shows example B-mode images of the pulsating RA of one volunteer, which was similarly observed for all volunteers, thus providing confidence of the RA location. After RA localization, PA data were acquired by illuminating the tissue using optical wavelengths of 750 nm, 810 nm, and 870 nm. For each wavelength, a total of 49 frames were acquired and averaged prior to image reconstruction.

Two beamformers were applied to reconstruct the photoacoustic images: (i) conventional amplitude-based using a single-step fast Fourier transform (FFT) reconstruction technique [45], and (ii) a coherence-based technique obtained with SLSC beamforming [32]. FFT reconstruction was performed using the k-Wave Matlab toolbox [45] and SLSC PA images were generated using the following equations [31], [32]:

(2) $$\overset{ˆ}{R}\left(m\right)=\frac{1}{N-m}\overset{N-m}{\underset{i=1}{\sum }}\frac{\overset{n_{2}}{\underset{n=n_{1}}{\sum }}s_{i}\left(n\right)s_{i+m}\left(n\right)}{\sqrt{\overset{n_{2}}{\underset{n=n_{1}}{\sum }}s_{i}^{2}\left(n\right)\overset{n_{2}}{\underset{n=n_{1}}{\sum }}s_{i+m}^{2}\left(n\right)}},$$

(3) $${\mathrm{SLSC}}_{\mathrm{pixel}}=\overset{M}{\underset{m=1}{\sum }}\overset{ˆ}{R}\left(m\right),$$

where N is the number of receiving elements, m is the lag (i.e., element distance) between two elements, $s_{i}\left(n\right)$ is the zero-mean time-delayed signal received by the ith transducer element at the depth *n* in units of samples, $n_{1}$ to $n_{2}$ is the correlation kernel size, and $\overset{ˆ}{R}\left(m\right)$ is the spatial correlation between the signals $s_{i}\left(n\right)$ and $s_{i+m}\left(n\right)$ for a given lag m. To obtain a SLSC image, Eqs. (2), (3) were applied for all axial and lateral positions in the imaging region, computed using a correlation kernel size of 1.3$\lambda_{c}$ and M = 12 (based on theoretical predictions of coherence lengths for the range of expected target sizes for our application [31]). When displaying the photoacoustic images, pixel values were first normalized by the brightest signals in the PA or SLSC images from the volunteer with the darkest skin tone to provide the same reference for all images created with the same beamformer when discussing qualitative comparisons.

### 2.4 Volunteers

PA data from a total of 18 male volunteers were acquired for this study. The skin tone of the volunteers was classified as follows: light (n = 2), intermediate (n = 4), tan (n = 5), brown (n = 4) and dark (n = 3). The ITA° values, skin tone category, age and body mass index (BMI) of each volunteer are shown in Table 2. These experiments were conducted with approval from the University of Sao Paulo Research Ethical Committee (CAAE: 08860819.4.0000.5407).

**Table 2:** Volunteer information.

Volunteer	Age [years]	BMI [kg/m${}^{2}$]	ITA°	Skin tone
1	26	26.23	46.44	Light
2	21	17.53	42.68	Light
3	20	19.88	38.07	Intermediate
4	30	24.86	32.41	Intermediate
5	21	19.15	30.26	Intermediate
6	30	25.43	29.18	Intermediate
7	29	23.62	24.36	Tan
8	29	27.12	19.73	Tan
9	39	26.88	16.87	Tan
10	53	24.75	14.22	Tan
11	30	21.78	12.39	Tan
12	34	23.41	6.85	Brown
13	21	17.24	−3.34	Brown
14	23	25.13	−6.43	Brown
15	41	23.24	−6.53	Brown
16	40	34.60	−33.57	Dark
17	28	24.76	−46.79	Dark
18	38	26.64	−53.74	Dark

### 2.5 Quantitative analyses

To quantify the impact of melanin content and beamforming technique on the PA image quality, the SNR of the RA at the distal forearm was calculated. To better understand the influence of the signal amplitude and clutter level on the SNR results, three additional image quality metrics were evaluated: (i) clutter artifact level, (ii) RA signal, and (iii) skin signal. These four metrics were calculated for both amplitude-based and SLSC PA images using the following equations:

(4) $$\mathrm{SNR}=20\cdot \log_{10}\left(\frac{S_{RA}}{\sigma_{0}}\right)\mathrm{[dB]},$$

(5) $$\mathrm{Clutterlevel}=20\cdot \log_{10}\left(\frac{S_{clutter}}{S_{RA_{maxITA}}}\right)\mathrm{[dB]},$$

(6) $$\mathrm{RAsignal}=20\cdot \log_{10}\left(\frac{S_{RA}}{S_{RA_{maxITA}}}\right)\mathrm{[dB]},$$

(7) $$\mathrm{Skinsignal}=20\cdot \log_{10}\left(\frac{S_{skin}}{S_{RA_{maxITA}}}\right)\mathrm{[dB]},$$

where $S_{RA}$, $S_{clutter}$, $S_{skin}$, and $S_{RA_{maxITA}}$ are the signal amplitudes averaged over a ROI defined around the RA, the background, the skin, and the RA of the volunteer with maximum ITA° at 870 nm (i.e., optical wavelength corresponding to maximum RA absorption and minimum skin absorption when compared to 750 nm and 810 nm), respectively. $S_{RA_{maxITA}}$ was chosen as the reference value to enable relative comparisons of all results, with consideration that this signal is surrounded by the least clutter and is therefore associated with the best possible signal. Two background ROIs were defined to compute clutter level (i.e., one proximal to the RA and other immediately underneath the skin ROI, where more significant levels of clutter artifact are expected in the PA image). The $\sigma_{0}$ term is the standard deviation from the background ROI proximal to the RA. All ROIs were equal in size and were manually positioned based on the location of the RA and skin in corresponding B-mode images to avoid selection of small blood vessels. The same ROI was employed for each pair of matched PA and SLSC images per volunteer per wavelength. ROIs selection examples are provided in Supplementary Fig. S2.

### 2.6 Statistical analyses

The dependent variables of SNR, clutter level, skin signal, and RA signal were found to be normally distributed using the Kolmogorov–Smirnov test when stratified by beamformer (i.e., amplitude-based or SLSC). Mixed effects and generalized linear mixed models regression analyses were used to compare the dependent variables across the fixed effects of skin tone or wavelength while controlling for the repeated measures within subject. All tests were two-sided and significance was set at p<0.05. No adjustment was made for multiple comparisons. These analyses were performed using SAS version 9.4 (SAS Institute, Cary, NC, USA). In addition, a correlation analysis was performed to measure the extent to which (1) RA signal and RA SNR and (2) skin PA signal and clutter level are linearly related for each beamformer. This analysis was performed using MATLAB software (Natick, MA, USA).

## 3 Results

**Fig. 2:**
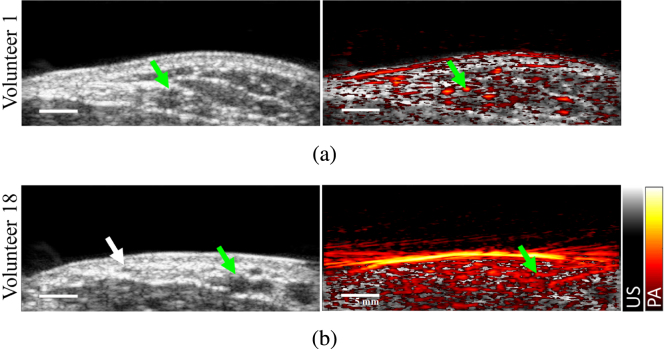
Ultrasound B-mode images (left) and B-mode overlaid by conventional amplitude-based PA image at 810 nm (right) of (a) volunteer 1 (lightest skin tone) and (b) volunteer 18 (darkest skin tone). The green arrows indicate the RA location. The white arrow indicates the location of a smaller blood vessel visible in the ultrasound B-mode image.

Fig. 2 shows two examples of ultrasound B-mode images (gray scale, left) and B-mode overlaid with color-encoded amplitude-based PA images acquired with 810 nm wavelength (right) for Volunteers 1 and 18 (lightest and darkest skin tones, respectively). The RA appears as the hypoechoic structure in the B-mode images, indicated by each green arrow. The PA image of the RA of Volunteer 1 is acceptable, with good visualization of the RA and surrounding small blood vessels (Fig. 2(a), right). However, the PA image of the volunteer with darker skin tone (i.e., Volunteer 18) contains strong clutter artifacts due to strong optical absorption at the skin surface, which compromises RA visualization (Fig. 2(b), right).

**Fig. 3:**
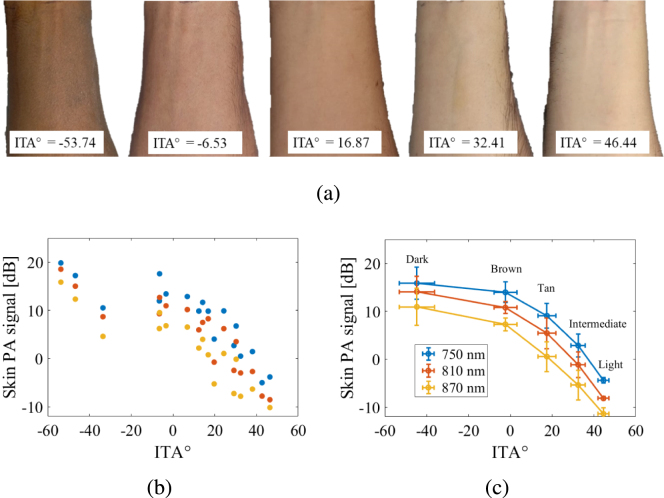
(a) Photographs of the forearm of five volunteers and the corresponding measured ITA° values, displaying dark to light skin tones (from left to right, respectively). (b) Skin PA signal as a function of ITA° for each volunteer, color-coded by optical wavelength. (c) Mean ± one standard deviation ITA° and skin PA signals, grouped by skin tone category and optical wavelengths.

Fig. 3 relates skin tone to quantitative ITA° values. Photographs of the forearm of one individual from each skin tone category and the corresponding ITA° values are shown in Fig. 3(a). Skin PA signal level as a function of ITA° for each volunteer and for the three wavelengths investigated are reported in Fig. 3(b), demonstrating that the skin PA signal is proportional to epidermal melanin content. This observation is consistent with the fact that darker skin tones contain higher levels of melanin concentration [29]. Volunteers were grouped by skin tone category according to their ITA° using the classification described in Table 1, resulting in Fig. 3(c), which shows the averaged ITA° for each skin tone category, independently reported for wavelengths 750 nm, 810 nm, and 870 nm. This result demonstrates that skin PA signal depends on both epidermal melanin content and optical illumination wavelength. Although the entire dataset suggests a nonlinear dependence on skin PA signal intensity with ITA°, a strong linear relationship (r = −0.8) was observed for the data of volunteers from light to brown skin tone range (volunteers 1 to 15). The nonlinear dependence on skin PA signal intensity with ITA° for the dark skin tone is particularly prominent with the dB scale used to display these results.

**Fig. 4:**
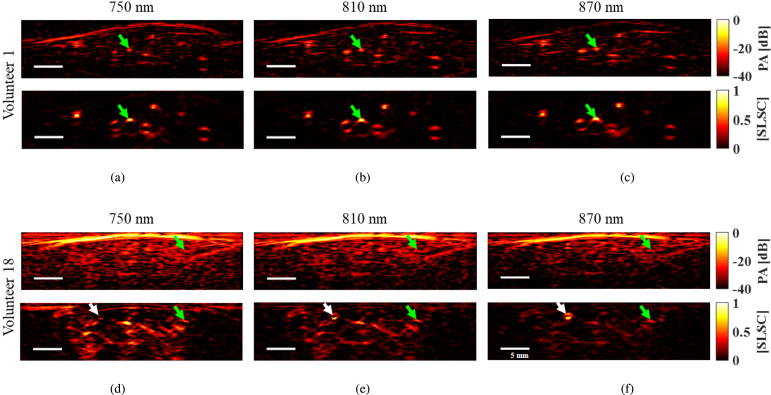
Amplitude-based (top) and SLSC (bottom) PA images of volunteer 1 (light skin tone) at (a) 750 nm, (b) 810 nm, and (c) 870 nm. (d)–(f) Amplitude-based and SLSC PA images of volunteer 18 (dark skin tone) at 750 nm, 810 nm, and 870 nm, respectively. The green arrows indicate the RA location. The white arrows indicate the location of a smaller blood vessel enhanced with SLSC imaging.

Fig. 4 shows amplitude-based (top row) and SLSC (bottom row) PA images of Volunteers 1 and 18 (lightest and darkest skin tones, respectively), acquired at 750 nm, 810 nm, and 870 nm wavelengths. Each RA location (determined from co-registered US images) is indicated by green arrows. Qualitatively, the skin PA signal amplitude decreases with wavelength for both volunteers, which can be explained by the known decrease in the optical absorption coefficients of melanin as wavelengths increases in the near infrared region [18]. Minimal acoustic clutter is present in the conventional amplitude-based PA images of Volunteer 1 for the three wavelengths, and RA visualization is not compromised. However, strong clutter artifacts are present in the amplitude-based PA images of Volunteer 18, particularly when acquired at 750 nm wavelength. In this case, it is not possible to distinguish the RA from the background due to this significant level of clutter artifacts. Although clutter is lower in the image acquired with 870 nm wavelength for this volunteer, RA visualization remains compromised in the amplitude-based PA image when compared to that of Volunteer 1.

The SLSC PA images in Fig. 4 contain reduced clutter and better visualization of the RA when compared to the amplitude-based PA images. In addition, the SLSC PA images qualitatively have good visualization of the RA and a low level of clutter artifacts, for both the light and darker skinned volunteers. This observation is further supported by the SLSC PA images in Figs. 4(d) to 4(f) improving visualization of a small blood vessel (white arrow) previously masked by clutter in conventional PA images. The increase in the coherence from this target when wavelength increases from 750 nm to 870 nm suggests that the feature indicated by the white arrow is an artery.

**Fig. 5:**
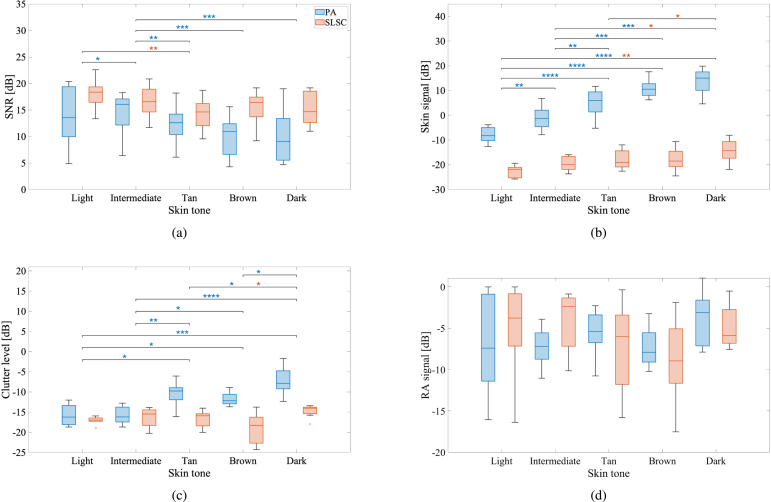
(a) SNR, (b) skin signal, (c) clutter level, and (d) RA signal calculated for the eighteen volunteers distributed among the various skin tone categories for the three wavelengths combined for amplitude-based (blue) and SLSC (red) PA images. The horizontal line inside each box displays the median. The upper and lower edges of each box represent the first and third quartiles. The vertical lines connected to the boxes show the minimum and maximum values in each group, excluding outliers, which are shown as dots and defined as any value >1.5 times the interquartile range. Statistically significant differences are indicated with *, **, ***, and **** denoting p<0.05, p<0.01, p<0.001, and p<0.0001, respectively.

Fig. 5 shows SNR, clutter level, RA, and skin signal amplitudes, calculated for the eighteen volunteers distributed among the various skin tone categories with results from the three wavelengths combined. Fig. 5(a) demonstrates a SNR decrease for darker skin tones for amplitude-based PA images (p<0.05), further highlighting the impact of skin melanin content on PA image quality. When SLSC beamforming was applied, an SNR improvement of 3.8 dB was achieved compared to conventional PA when considering the medians over all skin tones (p<0.05). In addition, the SLSC SNR achieved with the darkest skin tone was comparable to the amplitude-based SNR achieved with the lightest skin tone (i.e., p>0.05, indicating no statistically significant differences). Fig. 5(b) shows that skin PA signal increases with melanin content (p<0.05), which is supported by the qualitative observations in Fig. 4. Similarly, it is also consistent that the SLSC skin signal remains lower than the PA skin signal for all skin tones (p<0.05). Fig. 5(c) shows that the PA image clutter level increases with melanin content (p<0.05). Fig. 5(d) shows that the RA signal has overlapping values across the multiple volunteers (i.e., p>0.05 for both amplitude-based and SLSC images, indicating no statistically significant differences).

**Fig. 6:**
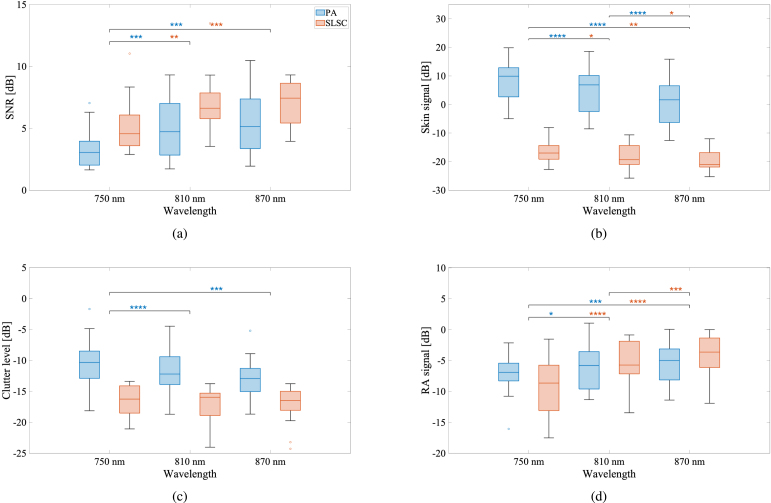
(a) SNR, (b) skin signal, (c) clutter level, and (d) RA signal calculated for the eighteen volunteers distributed among the wavelengths of 750 nm, 810 nm and 870 nm for all skin tone categories combined for amplitude-based (blue) and SLSC (red) PA images. Each box plot displays median, first and third quartile, minimum, maximum values (and outliers if present), as described in the caption of Fig. 5. Statistically significant differences are indicated with *, **, ***, and **** denoting p<0.05, p<0.01, p<0.001, and p<0.0001, respectively.

Fig. 6 shows SNR, clutter level, RA, and skin signal amplitudes, calculated for the eighteen volunteers distributed among the wavelengths 750 nm, 810 nm, and 870 nm for all skin tone categories combined. Fig. 6(a) shows that the RA SNR of the amplitude-based PA images increases with wavelength (p<0.05), which is consistent with the qualitative observations of Fig. 4. This observation is expected based on the known increase in optical penetration depth through skin and underlying tissue, combined with the known decrease of skin optical absorption, both occurring as wavelength increases from 750 nm to 870 nm [17], [18], [46], [47]. Also, consistent with qualitative observations, SLSC improved RA SNR when compared to amplitude-based PA for all wavelengths.

Fig. 6(b) shows that skin PA signal decreases as wavelength increases (p<0.05), which is expected based on the decrease in melanin optical absorption coefficients as wavelengths increase in the near infrared region [18]. Similarly, there is a decrease in SLSC values as wavelength increases (p<0.05), likely because of a disparity in coherent signals produced by the less optically absorbing melanin content at higher wavelengths. However, skin spatial coherence is more similar across the three wavelengths when compared to the decrease observed for the amplitude-based signals, likely because melanin concentration does not vary with wavelength. Therefore, the spatial coherence of melanin absorbers is not expected to significantly change as wavelength increases.

Fig. 6(c) shows that PA clutter level decreases as wavelength increases (p<0.05), likely because of the decreasing melanin absorption coefficient from 750 nm to 870 nm, as reported in [18]. The clutter level differences observed for the SLSC images as wavelength increases are not statistically significant (i.e., p>0.05), and the specific SLSC clutter values are lower than those of the amplitude-based PA images, likely due to the lower magnitude of the clutter source signals from skin (Fig. 6(b)).

**Fig. 7:**
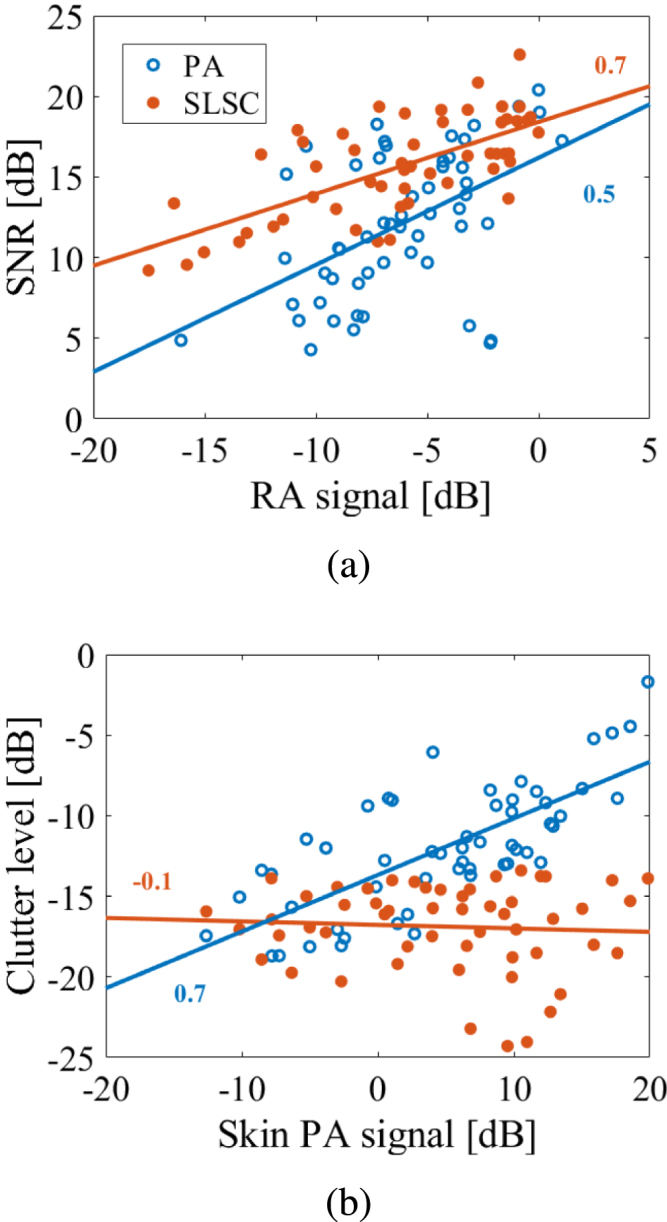
Correlation between (a) RA signal and RA SNR and (b) skin PA signal and clutter level for conventional amplitude-based PA imaging (blue) and SLSC beamforming (red).

Fig. 6(d) shows that the RA signal in both amplitude-based and SLSC PA images increased with wavelength (p<0.05). This PA signal improvement is likely due to the increase in optical absorption coefficient for oxygenated blood as wavelength increased from 750 nm to 810 nm [18]. The SLSC signal improvement is likely due to the decreased presence of acoustic clutter at the higher wavelengths (Fig. 6(c)).

Fig. 7(a) shows the correlation between RA signal and SNR for both amplitude-based and SLSC PA images. There is a relatively poor correlation (r = 0.5) between RA PA signal and SNR, likely due to the presence of acoustic clutter, which compromises the otherwise expected higher correlations between these two measurements. The correlation is greater (r = 0.7) between the RA SLSC value and SNR, which is more consistent with expectations that higher SNRs (i.e., lower noise caused by the presence of clutter) produce greater spatial correlations with the RA signal.

Fig. 7(b) shows clutter level as a function of skin PA signal amplitudes. There is a relatively strong correlation (r = 0.7) between the skin PA signal and clutter level, which quantitatively supports historical understandings that optical absorption at the skin surface is the source of this clutter [19], [21]. The correlation is lower (r = −0.1) between skin PA signal and SLSC clutter level, which is supported by the quantifiable clutter reduction achieved with SLSC beamforming (e.g., Figs. 5(c) and 6(c)).

## 4 Discussion

This work is the first to objectively assess skin tone and to both qualitatively and quantitatively demonstrate that skin PA signal and clutter artifacts increase with epidermal melanin content. Previous studies demonstrated that skin PA signal changes with epidermal melanin content and that darker skin tones impact PA images, creating clutter artifacts and deteriorating imaging quality [20], [22]. However, these studies employed a subjective classification of skin tone (e.g., FST scale), while our reliance on ITA° values to determine skin tone presents a more objective classification method. Del Bino et al. [30] demonstrated a linear relationship (R${}^{2}$ = 0.87) between ITA° and melanin index, which represents the fraction of the epidermis occupied by melanin. In addition, the skin optical absorption coefficient mainly depends on the epidermal melanin content [18], due to the elevated optical absorption of melanin, and skin PA signals are proportional to the skin optical absorption coefficient [1]. Therefore, the correlation of skin PA signal and ITA° observed in Figs. 3(b) and 3(c) is supported by existing studies.

While skin tone bias still exists to some extent in some SLSC PA images, particularly as the ITA° value decreases, as observed in Fig. 4, we focus our attention on the bias reduction achieved with SLSC relative to amplitude-based PA imaging. The two primary supporting results are captured by the SNR and clutter level measurements. First, the median SNR ranges 9.0–13.6 dB with conventional amplitude-based PA imaging, compared to 14.7–18.4 dB with SLSC PA imaging (Fig. 5(a)). Second, SLSC clutter levels were less impacted by melanin content when compared to conventional amplitude-based PA imaging (Fig. 5(c)). As skin tone darkened, median clutter levels increased by 8.3 dB (e.g., from −16.2 dB to −7.9 dB) for amplitude-based PA images, compared to only 3.0 dB (e.g., from −17.0 dB to −14.0 dB) for SLSC PA images. In addition, the associated median clutter level reduction by 0.7 dB for light skin tones (e.g., from −16.2 dB with amplitude-based PA imaging to −17.0 dB with SLSC PA imaging) was not as pronounced as the corresponding 6.1 dB reduction observed for dark skin tones in Fig. 5(c). A third supporting result is available in Fig. 7(b), which demonstrates a strong correlation between the clutter level and the skin PA signal in amplitude-based PA images that is absent from SLSC images, indicating that clutter is less influenced (i.e., less biased) by skin tone with SLSC imaging.

The quantifiable bias introduced by skin tone variations was successfully mitigated with SLSC beamforming (based on the measurements of RA SNR shown in Fig. 5(a)) with contributing factors that can be divided into optical considerations, spatial coherence influences, and clutter reduction benefits. Regarding optical considerations, while both the concentration of melanin absorbers and optical wavelength affect skin PA signal amplitude (Figs. 5(b) and 6(b)), melanin concentration appears to be more influential than optical wavelength when considering the impact on skin PA signal coherence. This observation is explainable from the perspective that melanin concentration varies with skin tone but not with wavelength. Regarding spatial coherence influences, an increase in melanin content likely creates more spatially coherent signals from skin, which increases the resulting clutter in the SLSC image (Fig. 5(c)). Conversely, images obtained with higher optical wavelengths, produce lower signal amplitudes from the skin, which translates to lower clutter levels (Fig. 6(c)). Regarding clutter reduction benefits, SLSC clutter levels were less impacted by melanin content when compared to conventional amplitude-based PA imaging (Fig. 5(c)). As skin tone darkened, median clutter levels increased by 8.3 dB for amplitude-based PA images, compared to 3.0 dB for SLSC PA images, as noted above based on the results in Fig. 5(c).

The clutter reduction achieved with SLSC imaging is related to skin signal amplitudes being consistently higher in conventional amplitude-based PA images when compared to corresponding signal magnitudes in SLSC images (Fig. 4, Fig. 5(b)). The higher absorption at the skin surface introduces more high-amplitude incoherent clutter that is not displayed in the corresponding coherence-based SLSC images (i.e., based on the design and purpose of this coherence-based beamformer). Because this clutter appears due to acoustic interactions that arise from optical absorption by the skin, simply removing skin PA signals after undesirable acoustic interactions have already occurred will not remove acoustic clutter from the corresponding images. In contrast, the lower spatial coherence of skin in the SLSC images can be explained by skin presenting as a broad target on a macroscopic scale, which produces short coherence lengths based on photoacoustic spatial coherence theory [31] and lower SLSC pixel values when implementing Eq. (3). From a molecular viewpoint, skin SLSC values increased with skin tone, likely because higher melanin content produces more optical absorbers, which results in more coherent signals from the skin and therefore higher skin SLSC values for darker skin tones.

There are a few counterintuitive observations based on the quantitative results presented herein. First, for darker skinned volunteers, reduced laser fluence within the imaging plane is conceptually expected to produce lower RA PA signals. This expectation directly follows from the initial PA pressure being proportional to light fluence [1] and because blood optical absorption coefficient is not directly dependent on skin tone. However, Fig. 5(d) shows that the RA PA signal is similar for all volunteers. This result is more intuitive from the perspective that the increased levels of clutter with increased melanin content (Fig. 5(b)) increasingly overwrite the “real” RA signal amplitude. Therefore, although the RA PA signal decreases due to decreased light fluence, the clutter level in the RA region increases, which yields a similar PA signal amplitude within the RA location for all skin tone categories.

Another counterintuitive observation is the decreased RA SLSC value with increased melanin content (Fig. 5(d)), with melanin absorption reducing optical fluence throughout the imaging plane. This observation presents an apparent inconsistency with previous reports stating that SLSC imaging performs well in low-fluence environments [33], [35], [36]. One explanation for this apparent discrepancy is the competing factor of acoustic clutter from the skin (Fig. 5(c)), which decreases RA spatial coherence and lowers RA SLSC values for volunteers with higher melanin content.

Results from the darkest skin tone category do not follow the trend described above, as the corresponding RA signal (Fig. 5(d)) is not the lowest among the volunteers. This seeming outlier likely occurs because of the considerable clutter level quantified in Fig. 5(c) and because of the poor target visibility qualitatively observed in the images from this group of darker-skinned volunteers (e.g., Fig. 2, Fig. 4). In addition, the skin tone classification scale in Table 1 was derived from previous literature [28], [43], in which the earliest known report [43] does not include a “dark” category (i.e., the darkest classification is brown). Recognizing that there are different shades of brown and that the quantitative SLSC results in Fig. 5 do not show substantial differences between “brown” and “dark”, it is probably sufficient to maintain the original classification of brown for this beamformer, rather than introducing the more subjective category of “dark” to further subdivide shades of brown. One justification for combining the objective SLSC results of the “brown” and “dark” categories for each plot in Fig. 5 could be that beyond a specific shade of brown, the presence of clutter masks the true RA signal to the extent that SLSC beamforming cannot recover the spatial coherence of an underlying structure.

Limitations of the presented approach to reducing skin tone bias by implementing SLSC beamforming include the inability of resulting coherence-based images to quantify amplitude-based image parameters (e.g., oxygen saturation). However, the presented approach has benefits for a target segmentation approach that can be used to extract regions of interest for amplitude-based image parameter measurements, similar to previous reports of SLSC imaging being implemented for segmentation in tool tracking [15] and spectral unmixing alternative [48], [49] applications. This topic will be the focus of future work. Two additional future directions include integrating SLSC beamforming with spectral unmixing techniques for potentially enhanced bias reduction and developing realistic phantoms that mimic various skin tones [23], [50] and corresponding clutter levels.

## 5 Conclusions

The work presented in this paper demonstrates that skin PA signals are proportional to epidermal melanin content, resulting in reduced light fluence throughout the imaging plane and increased clutter levels as skin tone increases from light to dark, due to an increase in skin optical absorption coefficient. The elevated clutter level and reduced light fluence for darker skinned individuals introduces a bias in PA imaging based on epidermal melanin content. Although conventional amplitude-based PA images for light skinned volunteers presented reasonable visualization of the RA, for darker skinned individuals, it was not possible to distinguish the RA from background due to the strong clutter level present in conventional PA images. This bias resulted in decreased SNR for darker skin tones. However, SNR was improved for all skin tones and wavelengths investigated when SLSC beamforming was applied. In addition, the SLSC images of volunteers with darker skin tones achieved comparable SNR to that obtained with a combination of light skin tone and conventional amplitude-based PA imaging. Therefore, SLSC beamforming successfully mitigates skin tone bias in PA imaging, resulting in images with good SNR and clear visualization of the RA for the range of skin tone categories investigated.

## Declaration of competing interest

The authors declare that they have no known competing financial interests or personal relationships that could have appeared to influence the work reported in this paper.

## Data Availability

Data will be made available on request.

## Appendix A Supplementary data

Video S1 Example B-mode images of the pulsating RA of one volunteer.

MMC S2 Figure S1 shows Phantom B-mode and PA images and stability results and Figure S2 shows ROIs selection used for the quantitative analyses.
