# Supplementary material for: Mitigating skin tone bias in linear array in vivo photoacoustic imaging with short-lag spatial coherence beamforming
Source: Photoacoustics. 2023 Sep 11;33:100555. doi: 10.1016/j.pacs.2023.100555 (PMC10658615; doi:10.1016/j.pacs.2023.100555)
Supplement: MMC S2 — Figure S1 shows Phantom B-mode and PA images and stability results and Figure S2 shows ROIs selection used for the quantitative analyses. [file mmc2.docx]

**Supplementary material**

**Mitigating skin tone bias in linear array *in vivo* photoacoustic imaging with short-lag spatial coherence beamforming**

Guilherme S. Pilotto Fernandes^a,b^, João H. Uliana^a^, Luciano Bachmann^a^, Antonio A. O. Carneiro^a^, Muyinatu A. Lediju Bell^b,c,d^, Theo Z. Pavan^a*^

^a^ Department of Physics, FFCLRP, University of Sao Paulo, Brazil

^b^ Department of Electrical and Computer Engineering, Johns Hopkins University, USA

^c^ Department of Biomedical Engineering, Johns Hopkins University, USA

^d^ Department of Computer Science, Johns Hopkins University, USA

*corresponding author

E-mail address: theozp@usp.br

**Keywords:** photoacoustic imaging, clutter artifact, melanin, short-lag spatial coherence, skin pigmentation, ultrasound, individual typology angle, Fitzpatrick scale

**Supplementary Figures**

Figure S1 - Phantom B-mode and PA images and stability results.

Figure S2 - ROIs selection

**SUPPLEMENTARY FIGURES**

**
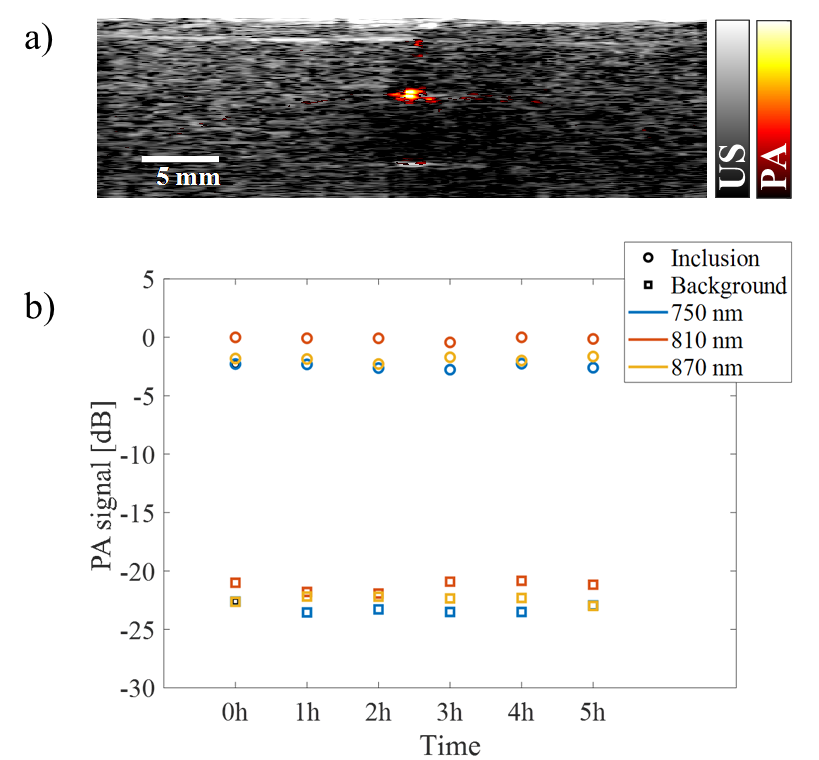
**

**Figure S1**. **a)** Ultrasound B-mode (gray) and PA image at 810 nm (color-encoded) acquired from a wall-less vascular phantom made using Styrene-ethylene/butylene- styrene (SEBS) copolymer-in-mineral oil as the base material. Glycerol was used to further adjust the acoustic properties and TiO_2_ to increase optical scattering. The vessel (4.5 mm in diameter) was filled with CuSO_4_ solution to mimic the optical absorption of blood. The optical absorption coefficients of the solution were 0.64 mm^-1^, 0.78 mm^-1^, and 0.73 mm^-1^ at 750 nm, 810 nm, and 870, respectively. **b)** Mean signal amplitude measured at hourly intervals, for ROIs selected around the vessel (circles) and phantom background (squares). Between each data acquisition, the transducer and coupling pad were removed from the phantom and repositioned in contact. PA signal magnitude remained stable throughout the analyzed period. Data were acquired using optical wavelengths 750 nm (blue), 810 nm (red), and 870 nm (yellow).


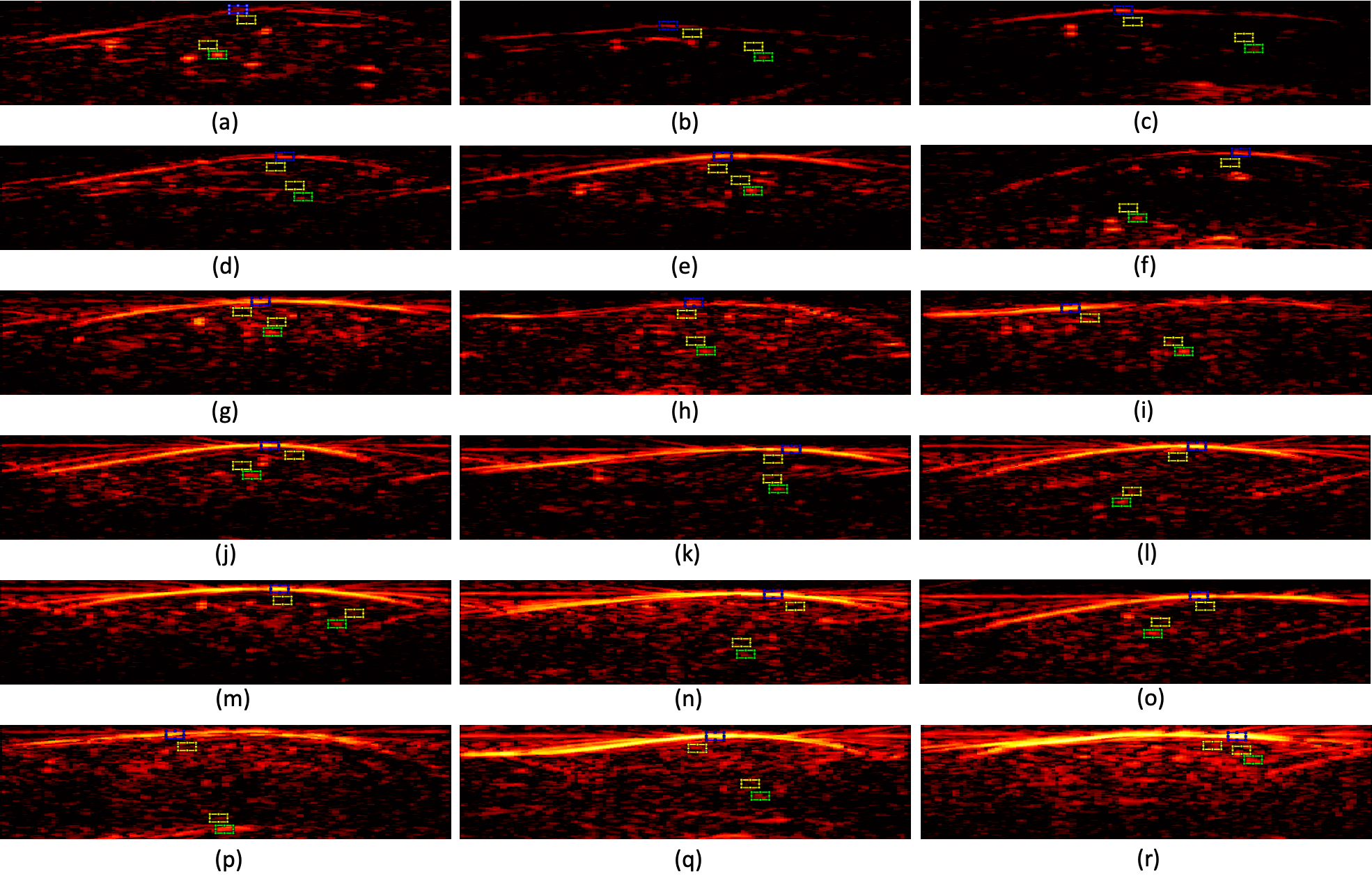


**Figure S2**. **a-r)** FFT PA images at 810 nm from volunteers 1 to 18, respectively, showing the ROIs used to compute the quantitative metrics. Four regions were selected, one around the radial artery (green), two on the background (yellow), and one on the skin (blue).
